# A robust edge-detection approach for precise delineation of deep structures from potential field data: Structural insights supported by remote sensing

**DOI:** 10.1038/s41598-025-23678-5

**Published:** 2025-11-04

**Authors:** Ahmed M. Eldosouky, Saada A. Saada, Sammar A. Allam, Ahmed Abd El-Gawad, Ahmed Henaish, Sara Zamzam

**Affiliations:** 1 Department of Geology, Faculty of Science, Suez University, P.O. Box: 43221, Suez, Egypt; 2 Canal High Institute of Engineering and Technology, P.O. Box: 8134805, Suez, Egypt; 3 Geophysics Department, Faculty of Science, Ain Shams University, Abbassia, Cairo, 11566 Egypt; 4 Department of Geology, Faculty of Science, Zagazig University, Zagazig, 44519 Egypt

**Keywords:** Edge detection, Magnetic and gravity interpretation, Geophysics, Structural and tectonic analysis, sinai, Planetary science, Solid Earth sciences

## Abstract

This study presents a unique Logsigm Function (LSF) filter for edge detection, established to improve the delineation of geological structures from potential field data. The filter’s performance was first validated using synthetic gravity and magnetic models simulating complex geological configurations with varying depths and contrasts. Results confirm the LSF’s high precision in resolving both shallow and deep structural boundaries, even in the presence of noise, while maintaining computational simplicity and ease of implementation. The method was then applied to real gravity and magnetic data from Northern Sinai, Egypt, a geologically complex region affected by extensional and inversion tectonics. To complement and validate the geophysical results, surface lineaments were extracted from enhanced remote sensing datasets, including Landsat8 OLI and ALOS PALSAR DEM imagery. The comparison between surface and subsurface trends revealed systematic vertical variations in structural orientations, highlighting the role of inherited basement faults and deformation decoupling. The LSF results successfully matched known structures and uncovered previously unrecognized lineaments, offering new insights into the tectonic architecture and basin evolution of Northern Sinai. The integrated approach demonstrates the value of combining advanced filtering techniques with remote sensing to achieve robust structural interpretations. The simplicity, stability, and high resolution of the LSF method make it a powerful tool for structural geology, tectonic analysis, and resource exploration in complex geologic terrains.

## Introduction

Interpreting the Earth’s subsurface structure from potential field (PF) data, such as gravity and magnetic anomalies, presents inherent challenges despite its non-invasive and cost-effective advantages^1–4^. The primary difficulty stems from the ambiguity of potential fields: a given anomaly can be caused by multiple source distributions, making unique structural interpretations complex. For instance, both shallow, small-scale features and deep, large-scale structures can produce similar long-wavelength anomalies. Furthermore, noise, data resolution limitations, and the superposition of anomalies from various geological sources often obscure subtle structural discontinuities, geological boundaries, intrusions, and faults, which are critical for understanding subsurface architecture and identifying economically significant features like ore bodies, tectonic plate boundaries, and hydrocarbon traps^5–8^. Robust edge detection approaches are thus elementary tools in interpreting these data, facilitating geoscientists to outline these structural discontinuities where abrupt lateral dissimilarities in rock magnetic susceptibility or density occur^7–11^.

The enhancement approaches play a significant role in accurately delineating geological lineaments and structures, which are crucial for understanding regional tectonic controls^12–15^. Broad techniques are known to enhance the contained information in PF data^16–20^. These methods are predominantly based on the derivatives of PF (gravity and magnetic) anomalies^21–28^.

The earliest attempts to outline source edge locations involved using the zero values of the vertical derivative (VD), as demonstrated by^29^. Subsequently^30^, showed that the ultimate amplitudes of the total-horizontal gradient (THG) could effectively trace source borders. This technique has been successfully employed in numerous investigations^31–35^ to improve the detection of structural features. Another widely utilized filter is the analytic signal (AS), initially presented by^36^ for 2D sources and extended by^37^ for grid data. The AS has been widely applied in many studies to reveal subsurface structures^35,38–40^.

Further advancements include the tilt-angle (TA) approach, generated by^41^, which operates on the ratio of the vertical derivative to the THG to delineate boundaries^42^. further refined this by computing the THG of the TA (TDR_THDR) to detect source boundaries. To counteract anomalies with diverse amplitudes^43^, suggested the theta map (TM)^44^. introduced horizontal TA (TDX) and hyperbolic TA (HTA) to balance the amplitude of both small and large anomalies^45^. also introduced the balanced AS using orthogonal Hilbert transforms, and more recently^46^, an amplitude-balanced filter for detecting PF data edges. Innovations continue with filters based on functions like the Elliott Function^47^ and the hyperbolic tangent function^48^ for mapping PF data edges^49^. applied a Laplacian of Gaussian Filter to study copper-bearing deposits in NW China. Notably^8^, integrated edges extracted from the Improved Logistic and Logistic functions of THG with remote sensing and field studies to investigate the tectonic evolution and mineralization of the Safaga-Semna shear belt in Egypt, highlighting the growing trend of multidisciplinary approaches.

Furthermore, the Modified Non-Local Means (MNLM) technique proposed by^50^, improves noise reduction by incorporating unweighted Euclidean distance and Integral Image strategies, reducing parameter-tuning complicatedness and accelerating weight calculation compared to traditional NLM. It is notably effective for mitigating noise amplified by high-order gradients. More recently^51^, introduced an enhanced version of THG using a modified Gudermannian Function. The modified Horizontal-Gradient Amplitude (MHGA) approach^52^ is a high-order gradient-based boundary detector that refines boundary delineation and structural mapping by enhancing the resolution and stability of horizontal gradient-based filters. Their investigation addresses the intrinsic limitations of classical horizontal gradient approaches, particularly in noisy settings, through MNLM.

While potential field data provide invaluable insights into subsurface density and magnetic susceptibility variations, they intrinsically lack direct surface expression. This gap is effectively bridged by remote sensing (RS) data, which offer a powerful means to delineate surface lineaments, fault traces, and other geomorphological features directly related to underlying geological structures. Satellite imagery analysis techniques such as multispectral products (e.g., Landsat, Sentinel2), radar data (e.g. Sentinel1), high-resolution commercial imagery (Google Earth Pro), and digital elevation models (DEMs) enable the mapping of subtle topographic expressions, drainage patterns, textural, and relief contrasts that often correspond to zones of weakness, faulting, or lithological contacts. The ability of RS to provide a regional overview of surface geology and its structural framework is indispensable^53–57^.

The true power in understanding the overall architecture of complex geological settings, particularly sedimentary basins, lies in the synergistic integration of remote sensing data with potential field data. Remote sensing helps constrain and validate interpretations derived from potential fields by providing surface evidence for buried or obscured structures. For example, a linear magnetic anomaly might be confidently interpreted as a fault if it aligns perfectly with a linear drainage pattern or a topographic escarpment identified through RS. This integration allows for a more holistic and robust understanding of the subsurface, linking observed geophysical anomalies to tangible surface expressions. It helps mitigate the inherent ambiguities of potential field methods by providing independent, complementary information, thereby improving the precision in delineating both shallow and deep structures and ultimately leading to more reliable tectonic and economic interpretations.

This study introduces the Logsigm Function (LSF) filter for edge detection that incorporates computational easiness, precision, and enhanced performance. Unlike many existing filters, the proposed LSF filter is designed to delineate both surface and subsurface features from PF data accurately, even in the presence of noise or complicated source settings. The LSF filter will be rigorously evaluated on synthetic models with overlapping gravity and magnetic sources to simulate real-world geological conditions. Moreover, it will be applied to real PF data from Northern Sinai, Egypt, to map both shallow and deep structures and study the Syrian Arc system^58^. The results will then be integrated with remote sensing data and other available geological information to introduce a new, reliable tectonic model for the study area, contributing significantly to the understanding of this complex region.

**Fig. 1:**
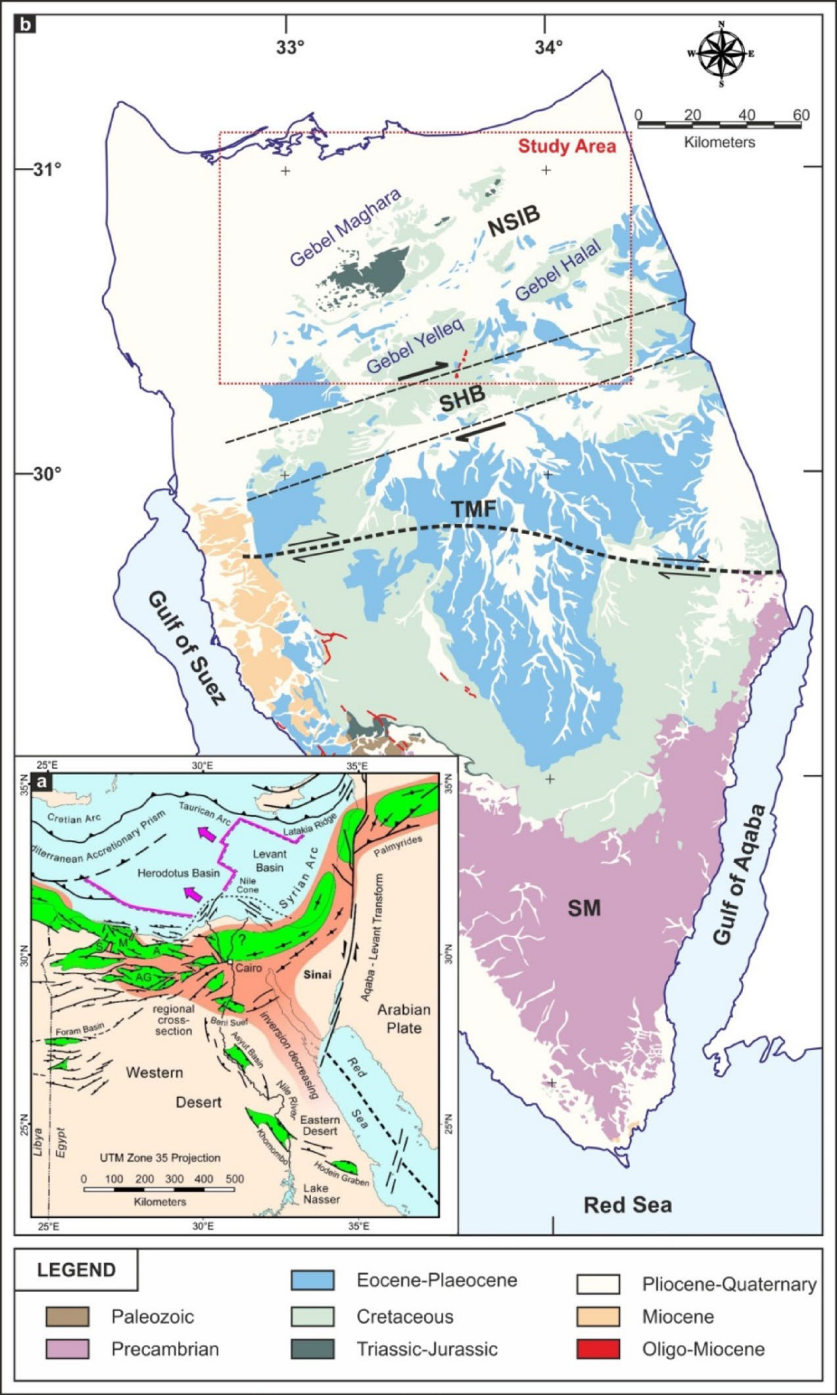
(**a**) Regional geological framework of northeastern Africa and the eastern Mediterranean, modified from^61^. A: Alamein Basin; AG: Abu Gharadig Basin; M: Matruh Basin; S: Shushan Basin. The direction of Triassic rifting and the boundary between Neotethyan oceanic and continental crust are based on^62^. (**b**) Geological map of Sinai showing the main structural systems and the distribution of rock units, modified after^63^. (Generated by CorelDraw X5, https://www.coreldraw.com).

The Syrian Arc system^58^ represents a broad regional tectonic framework characterized by Late Cretaceous-Cenozoic inversion structures that extend from Syria through the Eastern Mediterranean into northern Egypt (Fig. 1a). This system is a result of the northward convergence between the African and Eurasian plates and is marked by large-scale folds and reverse faults developed through the reactivation of earlier extensional faults. Within this regional context, the structural architecture of the Sinai Peninsula emerges as a complex interplay of tectonic influences owing to its position at the triple junction of the African, Arabian, and Eurasian plates (Fig. 1b). Situated at the northern end of the Red Sea Rift and bordered to the east by the Dead Sea Transform Fault system, Sinai has experienced successive episodes of rifting, compression, and strike-slip tectonics since the Pan-African Orogeny. The peninsula is broadly subdivided into several structural provinces, each reflecting a dominant tectonic style and distinct geologic history (Fig. 1b). Notable among these in northern Sinai are the Themed Fault^59^, the Sinai Hinge Belt^60^, and a series of Syrian Arc-related inversion folds, including three mountains (Gebels): Halal, Yelleg, and Maghara.

## New proposed logsigm function (LSF) filter

In recent years, several modifications of the horizontal gradient filter have been proposed to enhance edge detection in potential field data. For instance^64^, introduced the *logistic of the total horizontal gradient (LTHG)* method, which combines the ratio of the first vertical derivative to the total horizontal derivative with the logistic function. This approach allows both large- and small-amplitude edges to be highlighted simultaneously, which is defined as:

1 $$\:LTHG={\left[1+exp\left(-\frac{\frac{\partial\:THDR}{\partial\:z}}{\sqrt{\frac{\partial\:THDR}{\partial\:x}+\frac{\partial\:THDR}{\partial\:y}}}\right)\right]}^{-\alpha\:}$$

$\:\frac{\partial\:THDR}{\partial\:x}$, $\:\frac{\partial\:THDR}{\partial\:y}$ and $\:\frac{\partial\:THDR}{\partial\:z}$ are the x, y and z gradients of the THDR of the potential field data F.

And THDR is defined by:

2 $$\:THDR\:=\sqrt{{\left(\frac{\partial\:F}{\partial\:x}\right)}^{2}+{\left(\frac{\partial\:F}{\partial\:y}\right)}^{2}}\:\:$$

where ∂F/∂x and ∂F/∂y are the derivatives in x and y direction of F.

More recently^52^, proposed the modified horizontal gradient amplitude (MHGA) technique, which employs an improved ratio of the first-order vertical and horizontal gradients of the horizontal gradient amplitude for gravity and RTP magnetic data. MHGA defined as:

3 $$\:MHGA=\frac{\left|R+1\right|-\left|R-1\right|}{2}$$

Where R defined as:

4 $$\:R=\left(\frac{\frac{\partial\:THDR}{\partial\:z}}{\sqrt{{\left(\frac{\partial\:THDR}{\partial\:x}\right)}^{2}+{\left(\frac{\partial\:THDR}{\partial\:y}\right)}^{2}}}-\frac{\pi\:}{3}\right)$$

Our study proposes a new robust filter based on the total horizontal derivative (THDR) introduced by^65^. The THDR filter remains one of the most widely applied traditional edge detectors, as it places maximum amplitudes directly above anomaly boundaries. The mathematical foundation of our method draws on the logistic function, which produces a sigmoidal (S-shaped) curve similar to the arctangent function frequently used for edge identification in potential field analysis. We used the pseudovertical derivative obtained from the wavenumber domain method.

The LSF filter is defined as:

5 $$\:LSF={\left(\frac{1}{1+\text{e}\text{x}\text{p}(-kernel\_R)}\right)}^{"{2+Range}"}$$

In contrast with Eq. (1), the term (2 + Range) constrains the method to a narrower scaling interval and makes it highly sensitive to localized features. The parameter kernel_R is defined as:“”

6 $$\:kernel\_R=\left[K\left(\frac{\frac{\partial\:THDR}{\partial\:z}}{\sqrt{{\left(\frac{\partial\:THDR}{\partial\:x}\right)}^{2}+{\left(\frac{\partial\:THDR}{\partial\:y}\right)}^{2}}}\right)\right]"-\pi\:/4\:"\:\:$$

As in Eq. (4), this phase shift adjustment modifies the balance between vertical and horizontal derivatives and therefore alters the way anomaly edges are emphasized. The parameter *K* was tested over a range of positive values from 0 to 12. The experiments showed that the most reliable and stable results were obtained when *K* was chosen within the interval 0.5 to 3 and the *Range* in Eq. (5) is real number between 0.5 and 1.5 that is defined by the researcher.

## Synthetic models

Initially, complicated synthetic gravity and magnetic models include several prisms situated at different locations varying in size and depths to assess the performance of the LSF method.

**Fig. 2:**
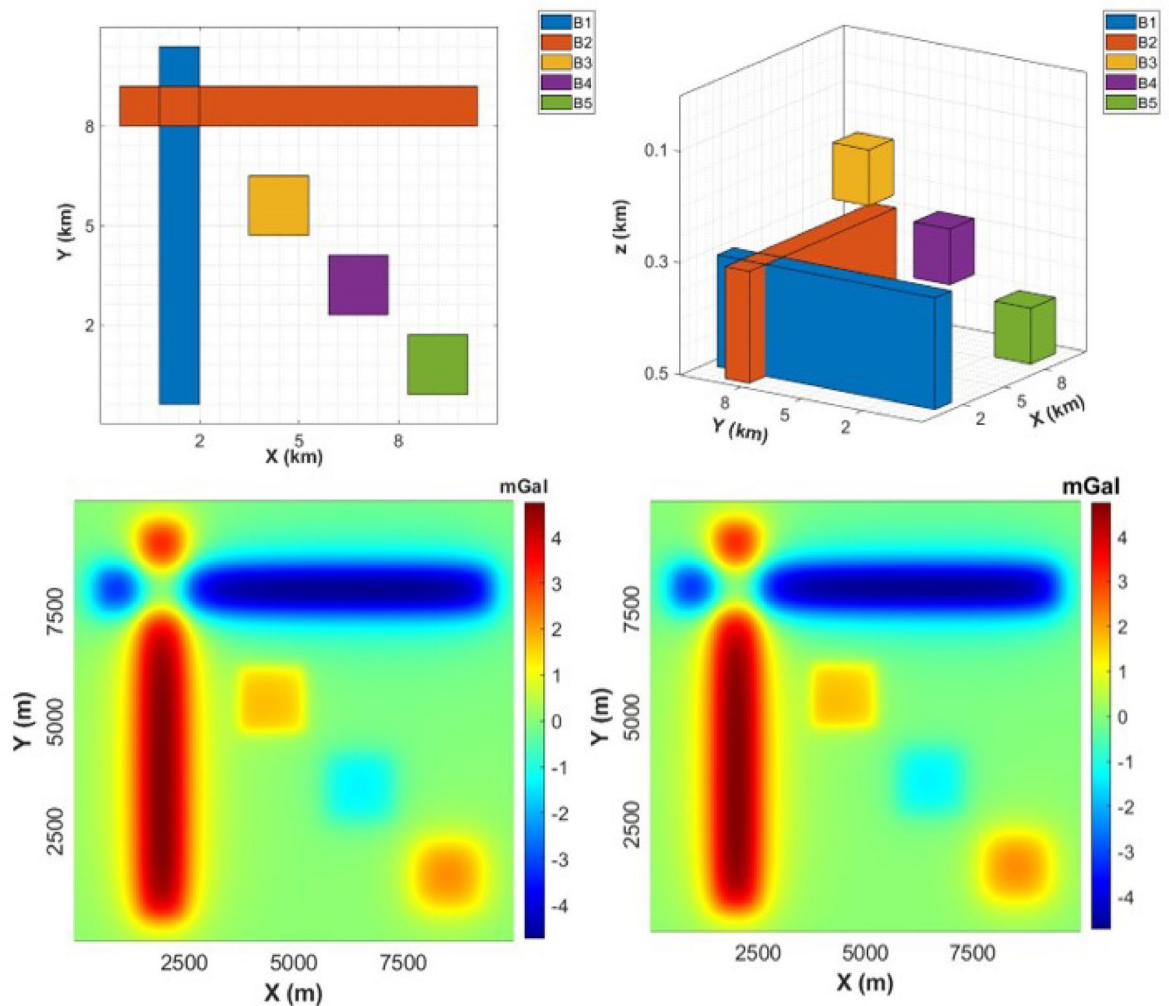
Synthetic gravity model: (**a**) Plane view, (**b**) 3D view, (**c**) Gravity anomaly, and (**d**) noise-gravity anomaly.

Figure 2a and b display the plane and 3D views of the gravity model that includes five sources. In this example, we consider a complex gravity model that contains two thin crossed and perpendicular sources (B1 and B2) and three prismatic sources superimposed at different depths (B3, B4, and B5) (Fig. 2). The parameters of this gravity example are presented in Table 1. Figure 2c illustrates the gravity anomaly induced by the model while Fig. 2d shows gravity anomaly with an applied noise of 0.002 mGal, which corresponds to approximately 0.05% of the maximum amplitude.

**Fig. 3:**
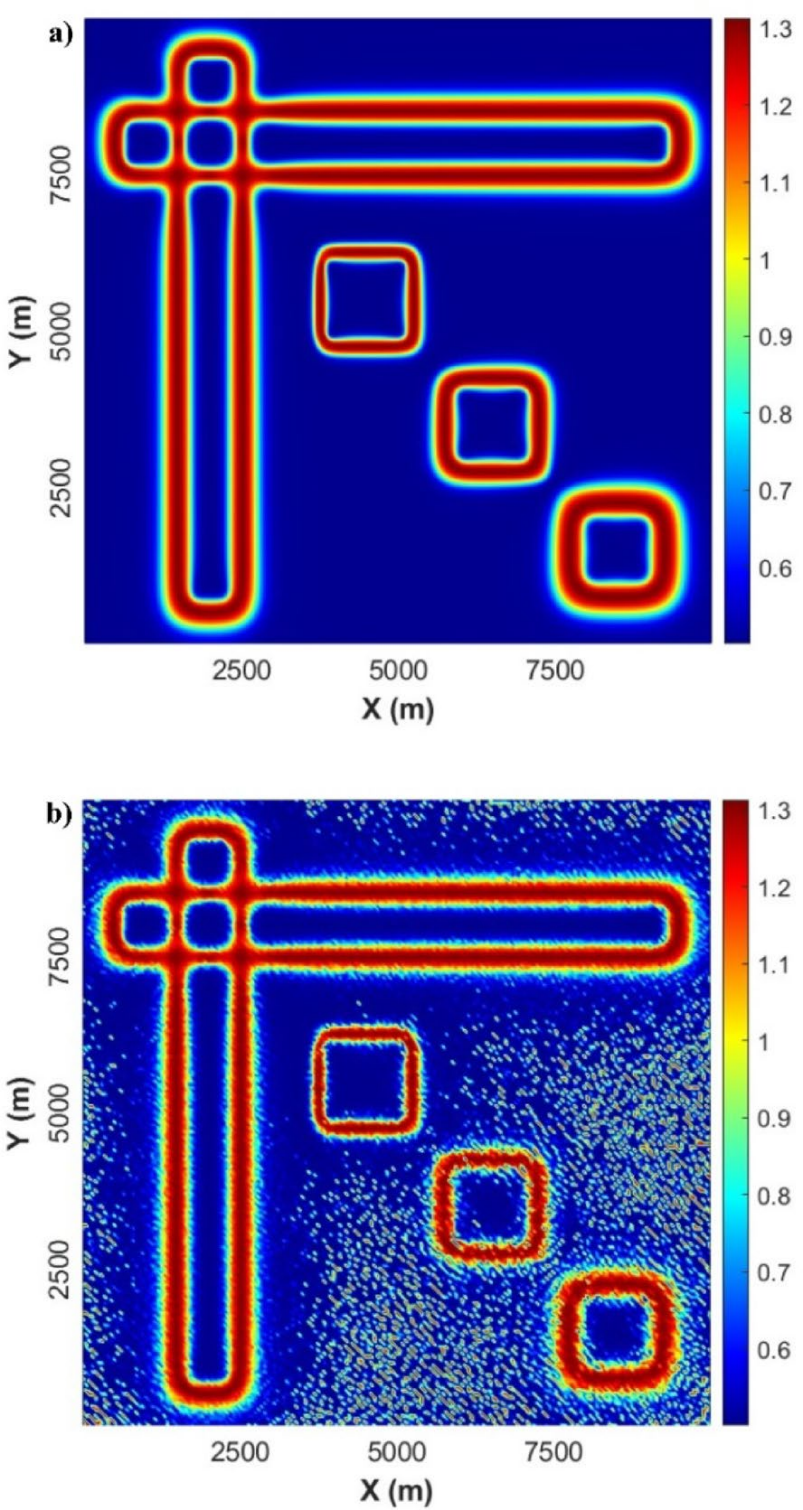
(**a**) LSF of gravity anomaly, and (**b**) LSF of noise-gravity anomaly.

Figure 3a displays the result obtained from the application of the LSF to the gravity-modelled data. It can be evidently noticed that the LSF can produce sharp and precise responses and detect all the source boundaries in a high resolution without generating any false edges. To further evaluate the stability of our new method, we applied the LSF to noise-gravity anomaly (Fig. 3b). It can be seen that has low sensitivity to the presence of noise. The LSF gives high-resolution edges of the source boundaries.

**Table 1:** The Spatial and physical parameters of the synthetic gravity model.

Parameters/model label	B1	B2	B3	B4	B5
x-coordinates of center (m)	2000	5000	4500	6500	8500
y-coordinates of center (m)	5000	8000	5500	3500	1500
Width (m)	1000	9000	1500	1500	1500
Length (m)	9000	1000	1500	1500	1500
Thickness (m)	200	200	100	100	100
Depth to top (m)	300	300	100	250	400
Inclination (°)	90	90	90	90	90
Declination (°)	0	0	0	0	0
Density contrast (kg/m³)	1000	−1000	500	−500	1000

**Fig. 4:**
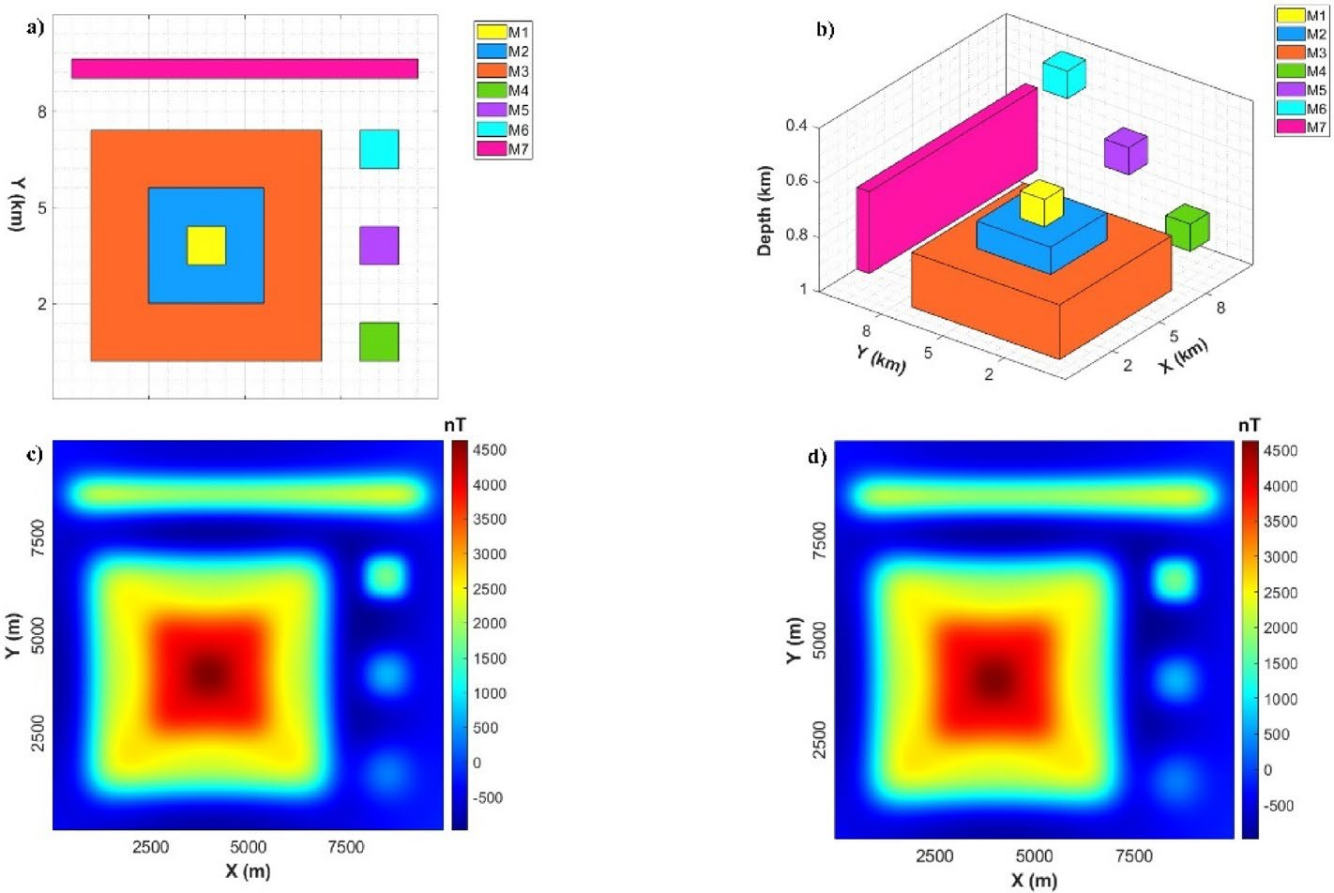
Synthetic magnetic model: (**a**) Plane view, (**b**) 3D view, (**c**) Magnetic anomaly, and (**d**) corrupted magnetic data with added random noise of 2%.

Figure 4 shows the magnetic anomaly model that contains seven magnetized prismatic sources (M1-M7). The nomenclature with a 2D representation of the magnetic model is presented in Fig. 4a and the schematic diagram of the 3D synthetic model considered for the present study is shown in Fig. 4b. The dimensions and physical properties of the seven prismatic sources are listed in Table 2. The theoretical magnetic anomaly is shown in Fig. 4c and corrupted magnetic data with added random noise having an amplitude of 2% of the anomaly amplitude is presented in Fig. 4d.

**Table 2:** The Spatial and physical parameters of the synthetic magnetic model.

Parameters/model label	M1	M2	M3	M4	M5	M6	M7
x−coordinates of center (m)	4000	4000	4000	8500	8500	8500	5000
y−coordinates of center (m)	4000	4000	4000	1500	4000	6500	8600
Width (m)	1000	3000	6000	1000	1000	1000	9000
Length (m)	1000	3000	6000	1000	1000	1000	500
Thickness (m)	100	100	200	100	100	100	600
Depth to top (m)	600	700	800	700	450	100	300
Inclination (°)	90	90	90	90	90	90	90
Declination (°)	0	0	0	0	0	0	0
Magnetic susceptibility (kg/m³)	0.5	1.3	1.5	1	−1	1	−1.1

**Fig. 5:**
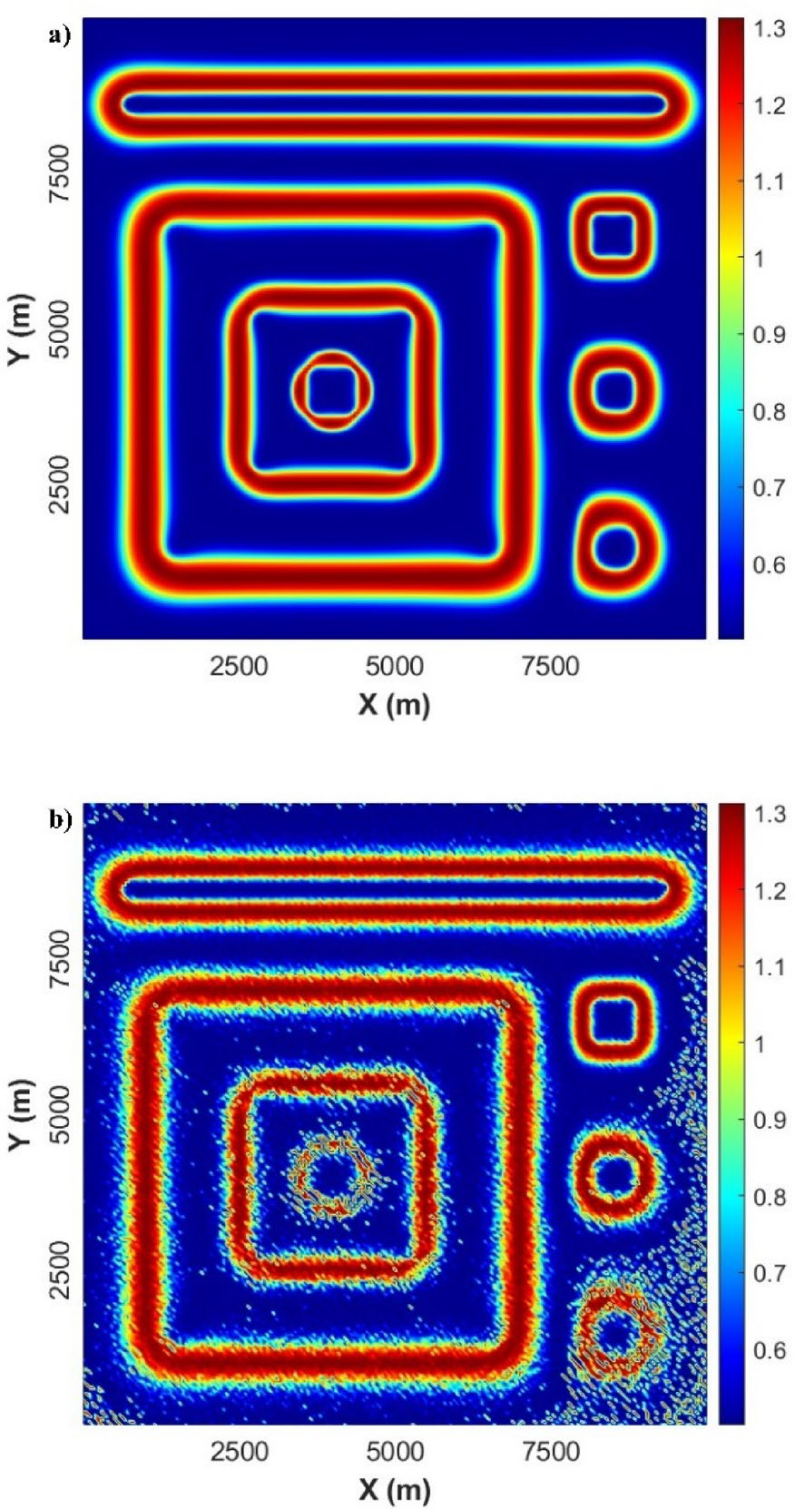
(**a**) LSF of magnetic-modelled data, and (**b**) LSF of the noise-corrupted magnetic anomaly.

Figure 5a shows the effect acquired from applying the LSF filter to the magnetic-modelled data. Like in gravity results, it can be clearly noticed that the LSF can produce precise and sharp for all edgers and boundaries of various magnetic sources in a high resolution without causing any incorrect boundaries. Figure 5b displays the LSF of the noise-corrupted magnetic anomaly. The outcomes obtained from the usage of the LSF show successful results in extracting the boundaries of all seven sources with high accuracy and can avoid inducing spurious lineaments.

## Regional structural and tectonic setting of the study area

The Syrian Arc Fold Belt is a regional, Late Cretaceous–Early Cenozoic compressional belt extending from Syria through Palestine into Northern Sinai^58^. In Sinai, it is characterized by a series of NE-SW to ENE-WSW trending asymmetric folds, often associated with reverse faults and thrusts that formed due to the inversion of pre-existing normal faults^66^. These structures primarily reflect a compressional regime resulting from the convergence between the African/Arabian plates and Eurasia during the Late Cretaceous to Early Cenozoic^67^. The prominent anticlines of Gebel Halal, Gebel Yelleg, and Gebel Maghara are typical examples, representing significant structural highs formed through the inversion of Mesozoic rift basins within this fold belt^67^.

**Fig. 6:**
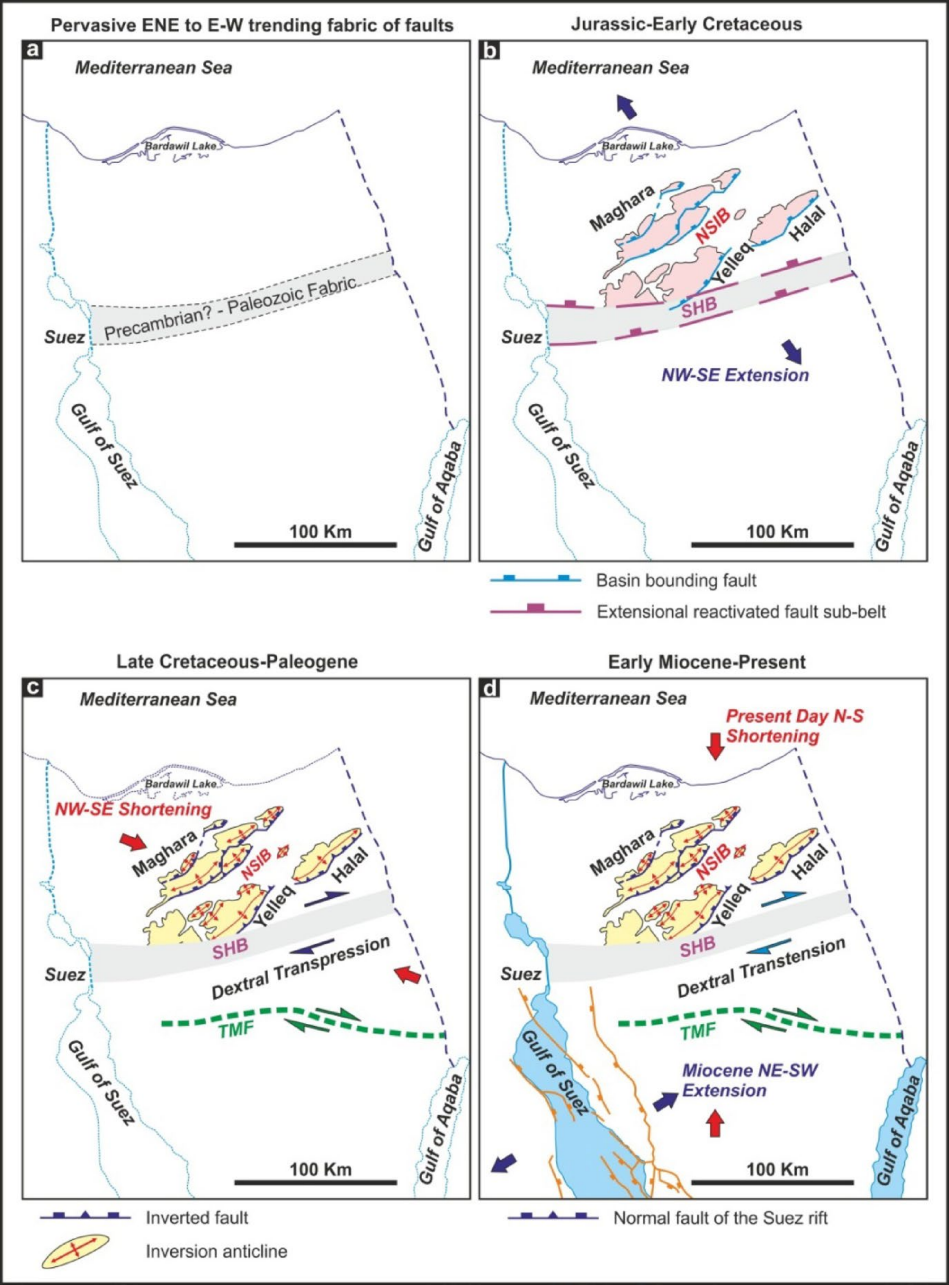
Proposed model illustrating the structural evolution of northern Sinai and adjacent regions, after^68^: (**a**) Pre-Mesozoic. Pervasive ENE–WSW to E–W trending fabric of faults. (**b**) Jurassic to Early Cretaceous: The reactivation of deep-seated basement structures led to extensional deformation along the Sinai hinge zone, resulting in the formation of half-graben basins across northern Sinai. (**c**) Late Cretaceous to Early Paleogene: The region experienced dextral transpressional tectonics along the hinge zone, which caused the inversion of the previously formed basins. (**d**) Early Miocene to the present day: Tectonic activity shifted to rifting processes in the Gulf of Suez, accompanied by dextral transtensional movements along the Sinai hinge zone. (Generated by CorelDraw X5, https://www.coreldraw.com).

The Sinai Hinge Belt represents another key structural element in northern Sinai. It is a fundamental, ancient crustal boundary characterized by a narrow, 20–25 km wide and 250 km long ENE–WSW trending structural zone that extends westward from the Dead Sea Transform system^68^. This belt acts as a critical boundary between two tectonically distinct domains: to the south lies a stable platform with a relatively thin Mesozoic sedimentary cover and shallow Precambrian basement, while to the north, the crust contains a thicker Mesozoic sequence and deeper basement rocks that host the heavily folded Syrian Arc structures. The Sinai Hinge Belt originated in the Precambrian or Paleozoic and (Fig. 6a) has undergone multiple phases of reactivation, initially as normal faults during early Mesozoic extension associated with the opening of the Neotethys Ocean, then as dextral transpressional features during Late Cretaceous–Early Tertiary Neotethys closure, and later as dextral transtensional structures during the Miocene. Its structural inheritance and reactivation history played a pivotal role in shaping the geometry and segmentation of the Northern Sinai inverted basins.

The inverted basins of Northern Sinai represent a crucial component of the Syrian Arc system, offering insights into the complex interplay between pre-existing extensional structures and subsequent compressional reactivation. An inverted basin is fundamentally a former extensional basin (graben or half-graben) whose bounding normal faults have been reactivated as reverse faults, leading to structural uplift and the formation of anticlines.

The deformation history of Gebel Halal, Gebel Yelleg, and Gebel Maghara exemplifies this inversion process. During the Jurassic and Early Cretaceous, Northern Sinai experienced a period of regional NW–SE oriented extension (Fig. 6b), related to the incipient opening of the Neo-Tethys Ocean^68^. This extensional regime led to the development of numerous NW–SE trending normal faults and a series of localized half-grabens^69^. These basins served as depocenters for thick accumulations of Mesozoic sediments, including Jurassic and Early Cretaceous clastics and carbonates. The presence of these pre-existing normal faults created weakness zones that were later reactivated during inversion. Seismic profiles often show growth strata thickening towards these original faults, providing evidence for this extensional phase.

The primary inversion occurred during the Late Cretaceous to Paleogene (Santonian-Maastrichtian through Paleocene-Eocene), driven by a regional NE–SW compressional stress regime^68^. During this phase, the older normal faults were reactivated with reverse-sense motion, uplifting the previously deposited basin fill and forming the prominent anticlines that dominate the present-day landscape (Fig. 6c).

Following this major inversion, Neogene tectonics associated with the opening of the Gulf of Suez-Red Sea rift and the development of the Dead Sea Transform system caused limited reactivation of older structures or formation of new, smaller-scale features (Fig. 6d). Nevertheless, the main structural architecture established during the inversion remained largely intact^68^.

The deformation style in Northern Sinai’s inverted basins is strongly influenced by the mechanical properties and thickness of the overlying sedimentary succession, which rests unconformably on the Precambrian basement. The Mesozoic sequence, particularly significant in understanding the inversion, consists of Jurassic siliciclastic-carbonate units and thick Cretaceous sandstones, shales, and limestones. Overlying these, the Cenozoic succession includes Paleogene carbonates and Neogene clastics, mostly post-dating the main inversion phase.

The inversion process created folds and faults that are genetically linked to regional stress regimes and offer important surface indicators of deeper-seated structures. Understanding their geometry and orientation is essential for delineating both shallow and deep structural features using remote sensing and potential field techniques.

## Data and methodology

### Remote sensing data

Two remote sensed data were employed in the current study include Landsat8 Operational Land Imager/Thermal Infrared Sensor (OLI/TIRS) and Digital Elevation Model (DEM) from the Phased Array type L-band Synthetic Aperture Radar (PALSAR) data. Regarding their diverse abilities, both images were utilized to enable the identification of surface expressions of structural features through lithological boundaries mapping, distinct tonal, textural, and relief contrasts. The Landsat8 OLI/TIRS imagery, taken on May 25, 2025 (Path 175, Row 39), includes 11 spectral bands with different spectral and spatial resolution (visible, 15 m and infrared, 30 and 100 m). It was sourced from the USGS Earth Explorer platform and provided in UTM projection using the WGS 84 system. This image was calibrated and enhanced using radiometric and atmospheric corrections, as well as Gram-Schmidt spectral sharpening (by Panchromatic band) to enhance the quality of raw data and prepare them for analysis and processing techniques.

The enhanced Landsat8 image was clipped to the interested areas and utilized for distinguishing the lithological units and large-scale structural mapping through Principle component (PC), Minimum Noise Fraction (MNF), and Optimum Index Factor (OIF) analysis methods. PC and MNF are both dimensionality reduction techniques used in satellite image processing, but MNF includes a noise-whitening step that makes it more effective in isolating meaningful signals in noisy data. While PCA emphasizes data variance for enhancing major features, MNF improves the signal-to-noise ratio, making it more suitable for detailed geological and structural analysis^70–73^. The OIF is a powerful statistical tool in geological and structural mapping, as it identifies the most informative band combinations by maximizing spectral variability and minimizing redundancy^74–77^. This enhances the visual contrast between different rock types and structural features such as faults and fractures.

The PALSAR DEM with 12.5 m resolution, offered as part of the ALOS PALSAR Radiometrically Terrain Corrected (RTC) products, which is downloaded from the Alaska Satellite Facility (ASF) Distributed Active Archive Center (DAAC). Regarding to its effectivity in geological structural analysis^78^, this DEM was used to identify and automatically extract surface lineaments in our investigated area using the Directional Filtering (DF) method. This technique is a flexible image enhancement method commonly used to improve image sharpness, particularly for detecting edges and emphasizing linear structures. It is especially effective in highlighting geological features such as faults and fractures, thereby aiding in the extraction of lineaments. In the current study, convolution with 5 × 5 and 11 × 11 weighted kernels were applied to DEM to enhance sharp and broader features, respectively. Considering the previous geological studies, the filtering was performed in two specific directions NE-SW and NW-SE to enhance directional features through these dominant structural trends. Additional weighted sum step was operated using the resultant directional filtered images to reduce the apparent surface linear features that resulting from the wind’s effect on sand. Lineaments were automatically extracted from the resultant weighted sum image using the LINE tool in PCI Geomatica 2013 software (developed by the Canadian company PCI Geomatics, CATALYST Earth; https://catalyst.earth/customer-center/tutorials/) with various settings (Filter radius: 50; Gradient: 50; Length: 100; Line fitting error: 10; Angular difference: 30; Linking distance: 50). The analysis methods of Landsat8 OLI and PALSAR DEM were completed by ENVI, version 5.1 (generated by L3Harris.com, https://www.l3harrisgeospatial.com/Software-Technology/ENVI) and ILWIS (Developed by ITC, https://www.itc.nl/ilwis/) softwares, as well as the weighted sum tool and the final images were performed and exported using ArcGIS packages, version 10.3 (developed by esri.com, https://www.esri.com/en- us/arcgis/products/arcgis-desktop/overview/).

### Bouguer gravity (BG) data

The BG anomaly map of Sinai was compiled by two gravimeters (Scintrex CG-3 Autogravity micro-processor automated gravity meter and LaCoast Inc. Model). Both have accuracy of 0.01 mGal. The gravity Survey was at the same location and time of magnetic measurement points. Automatically by the Autograv unit, the recorded data includes station numbers, reading in mGal. Time at which station is measured. leveling of instrument. temperature, S.D (standard deviation) and error. These previous data are dumped by connection with PC Computer in the form or dump file. Text file was prepared includes station number, longitude, latitude and elevation. By using highly sophisticated programs^79^, the dump and text files were used in preparation of raw data files. The raw data file was used for data reduction (correction).

The data was reducted by appling Instrument drift correction which accounts for nonelastic chances in the instrument’s spring and temperature effects (the instrumental drift is less than 0.02mGal per day). The tide correction (effect of sun and moon) was applied. In addition, latitude correction was applied (the formula adopted by the International Association of Geodesy in 1967). The free air correction was applied by using the formula Flee air correction = −0.3086 h Where h is the elevation of measured station in meters with respect to sea level. Bouguer correction was applied using the formula Bouguer correction = 0.04193 Ϭ h Where Ϭ is density (2.67 gr.cm^−3^) and h is the elevation of measured station in meter with respect to sea level.

Finally, for topographic correction, a hill or mountain will exert an upward pull at the station and lend to reduce the gravity value at the station. So, a correction representing the hills attraction must be added to the measured gravity value at the station. Similarly, for depression the attention of the rock material needed to fill the depression, but this material does not exist. This attraction must again be added as a terrain correction to the measured value. The terrain correction required a detailed topographic map of area 1: 50,000 available. The terrain correction was done using chart of 5 km radius containing^80,81^. A concentric ring and a few radii drawn at suitable angular intervals. At the end, the processed data was gridded using a Kriging Algorithm with a grid cell size of 1000 m.

### Magnetic data

Total magnetic intensity field (nT) was measured with a magnetometer model G 816 as instrument with accuracy 1 nT. Diurnal variation of the earth’s magnetic field was measured by continuous recording base station with 1 min as a time interval to observe the earth’s diurnal activity. The stationary base station instrument is ENVI MAG magnetometer with 0.1 nT accuracy. The data processing was started by the diurnal variation removal. The allowed gradient of diurnal variation must not exceed than 10 nT within 10 min for the survey, according to the principle of base station establishment. It should be as close to or within the survey area and care must be taken in choosing the location of the base station. 24 base station locations were chosen for the magnetic survey for the whole of Sinai. The base station correction was applied to remove the effect of magnetic field variation (the corrected reading = the observed value - base reading). Daily accuracy of survey was calculated in the form of mean quadratic errors (η):

 

7 $$\eta=\:\pm\:\sqrt{\frac{\sum\:{d}^{2}}{2n}}$$

Where *d* is the difference in nT of repeated measurement point and *n* is the number of repeated points. The calculated accuracy of the whole magnetic survey is ± 3 nT. The IGRF was applied for each measured point.

## Results

### Surface data analysis (Remote sensing and structural mapping)

**Fig. 7:**
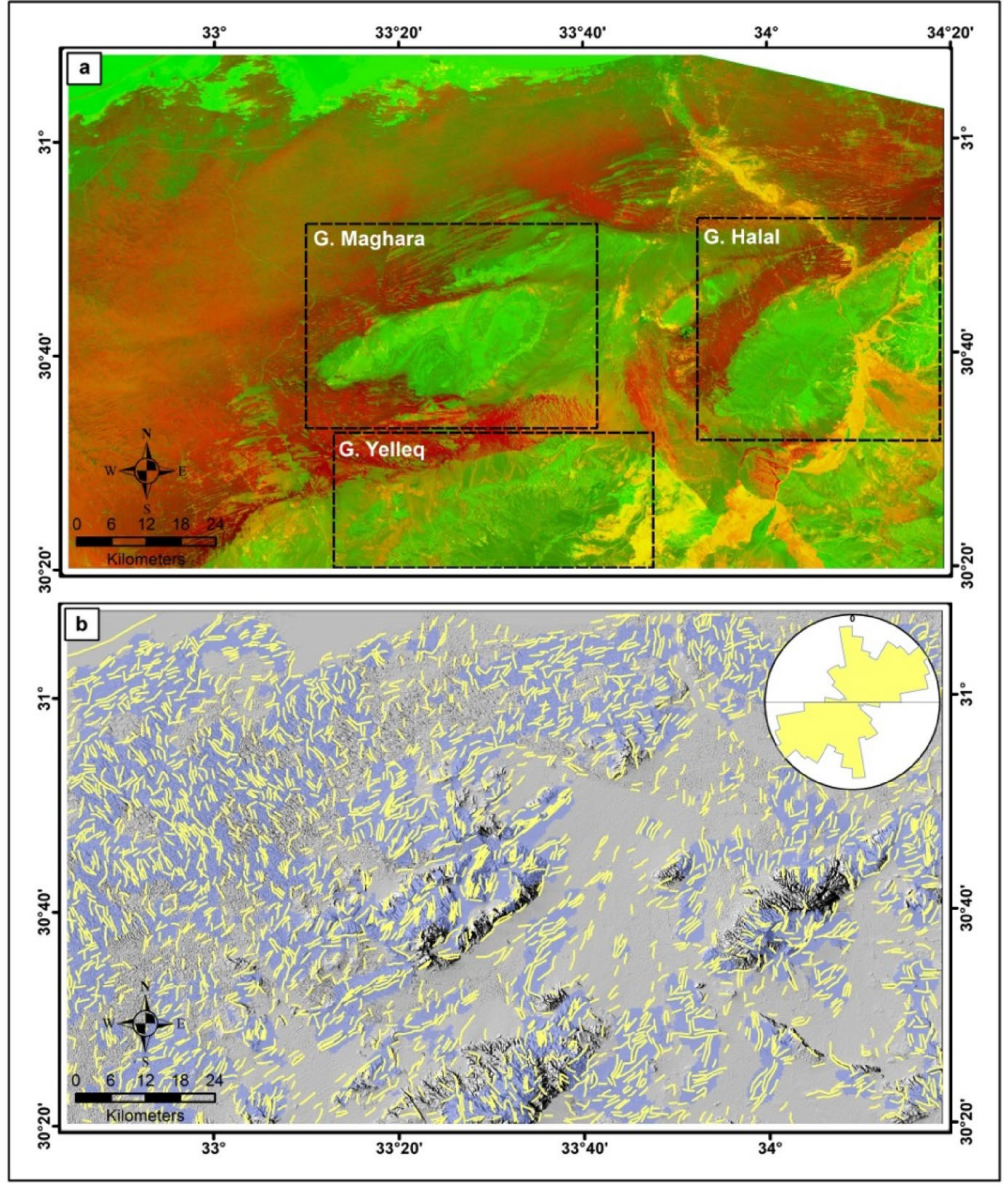
(**a**) False color composite image by RGB: PC1, MNF1, weighted sum DF, (**b**) Automatic extracted lineaments and their density overlaid on the weighted sum DF DEM image with their trend analysis as an inset. (Generated by PCI Geomatica 2013 software; https://catalyst.earth/customer-center/tutorials/, ArcGIS v.10.3. https://www.esri.com/ en- us/arcgis/products/arcgis-desktop/overview/, and RockWorks v.15 software; https://www.rockware.com/product/rockworks/).

The integration of the processed Landsat8 images (PC and MNF) with the weighted sum DF DEM image enhances the analysis and visual interpretation of extracted lineaments, offering precise, efficient, and qualitative results as shown in Fig. 7a. The false color composite image (R: PC1; G: MNF1; B: weighted sum DF DEM) emphasizes the uplifted geological units and their associated structures with shades of green color. However, the rest of the exposed deposits and their fluctuations resulting from hidden structures are indicated by shadows of red-green tonal contrast as evident in the northwest and west parts. Additional geomorphological linear features such as stream segments and terraces are observed with orange color. In general, the dominant surface appearance of the exposed geological units in the study area takes a north-easterly direction.

Figure 7b shows the automatic extracted lineaments from the weighted sum DF DEM image with 3061 lines. The trend analysis (rose diagram) of these lineaments indicates the predominance of NE-SW and N-S trends more than NW-SE, coinciding with the structural setting of the study area. The density analysis of these lineaments is very valuable as the higher density zones (blue color) are best matched with the real structures that are related to the uplifted geological units, the linear structures that are still preserved (in the southeastern part), and some of the vanished linear features in the blurred sand dunes regions (in the north and northwestern parts).

**Fig. 8:**
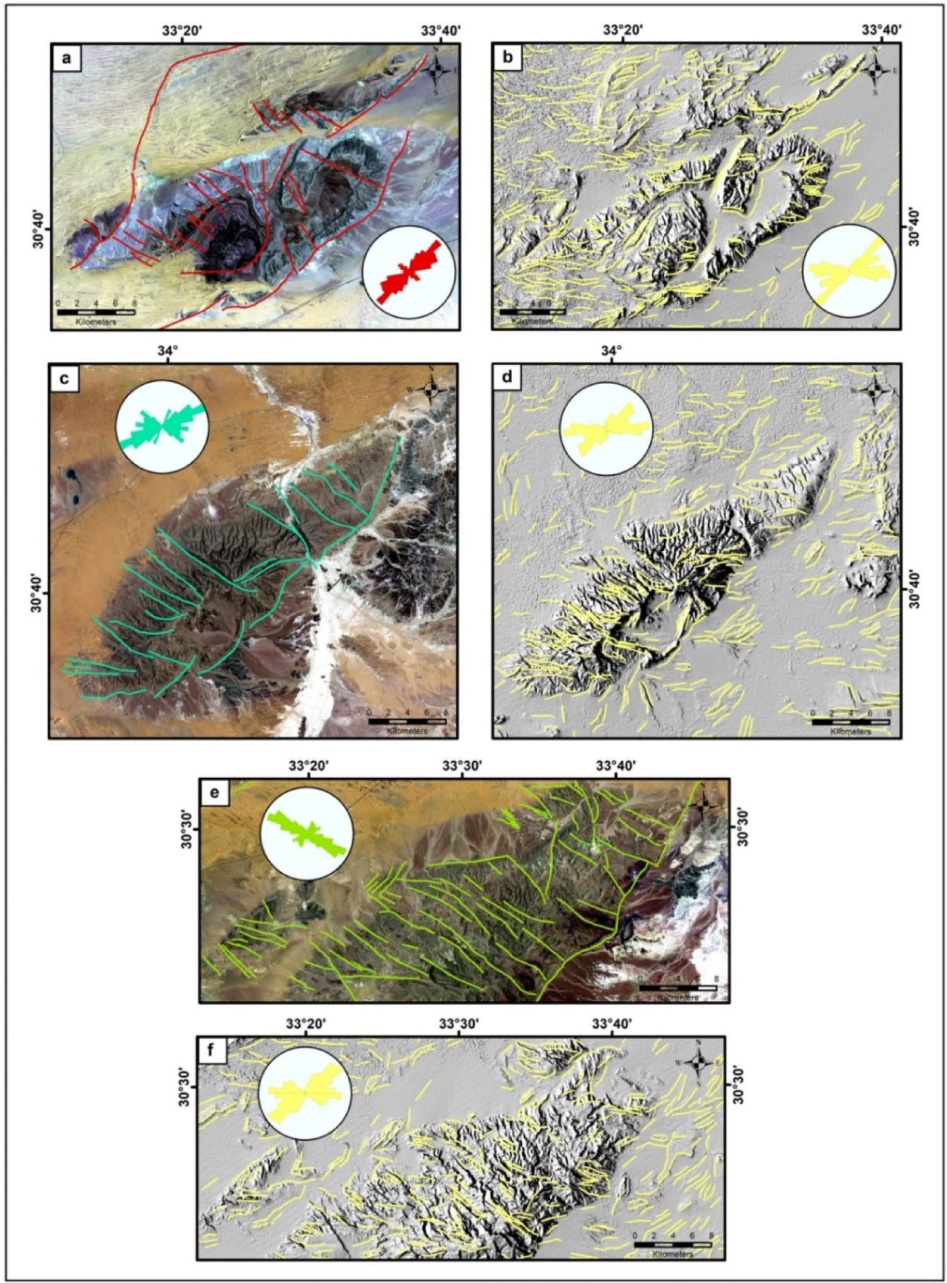
(**a**, **c**, **e**) RGB 741 L8 composite images for G. Maghara, G. Halal, and G. Yelleq, respectively, each of them overlaid by manually digitized structures, (**b**, **d**, **f**) Weighted sum DF DEM images for the three Gabals superimposed by automatically extracted lineaments (convolution with 5 × 5); Rose diagrams for manual and automatic interpreted lineaments are presented as Insets. (Generated by PCI Geomatica 2013 software; https://catalyst.earth/customer-center/tutorials/, ArcGIS v.10.3. https://www.esri.com/ en- us/arcgis/products/arcgis-desktop/overview/, and RockWorks v.15 software; https://www.rockware.com/product/rockworks/).

To evaluate the accuracy of the detailed automatic surface lineaments, we compared their distribution and directions with those of the manually mapped structures for the three exposed mountains (Gebels), which represent the regional tectonic setting of the study area (Fig. 8). The manual interpreted structures are visually delineated using OIF Landsat8 images and high spatial resolution Google Earth Pro images considering the previous geological studies^66,68^. Considering OIF results, we obtained Landsat8 741 bands as RGB composite images for the selected areas. Although the OIF index value of these bands as a composite image differs for the three regions (G. Maghara: first rank ~ 65.86; G. Halal: fifth rank ~ 59.23; G. Yelleq: third rank ~ 56.73), we used it to facilitate the visual comparison of the geological units of these mountains. It is evident that there is a similarity between the geological units exposed in G. Halal and G. Yelleq (Fig. 8c & e), as they have the same color shades that differ from G. Maghara (Fig. 8a). This is consistent with previous geological studies^66^, as G. Maghara is characterized by the presence of Jurassic rocks (dark blue color). Also, the optical images offer the best display for the linear and curved features, especially of G. Maghara as shown in Fig. 8a.

Regarding the correlation between the trend analysis results of automatically extracted and manually interpreted lineaments, there is agreement between the results for both G. Maghara and G. Halal, as the predominant trend is NE-SW and followed by E-W trend (Fig. 8a-d). However, there is a slight difference between the automatically extracted lineaments trend (NE-SW prevails) and the manually mapped ones (NW-SE predominant trend) in G. Yelleq as shown in Fig. 8e and f. It may be related to the over-statistics of the automatically extracted linear features surrounding it.

According to the distribution of surface lineaments, the number of automatically extracted lines is higher than the manually mapped ones, as expected. However, the manually delineated lineaments are more realistic, as the horizontal boundaries between the rock’s formations appear as linear features in the automatically extracted lineaments, as observed in G. Maghara. Therefore, it is necessary to adopt the results of the automatic extraction, but only after validating them through detailed geological studies.

### Potential data analysis

**Fig. 9:**
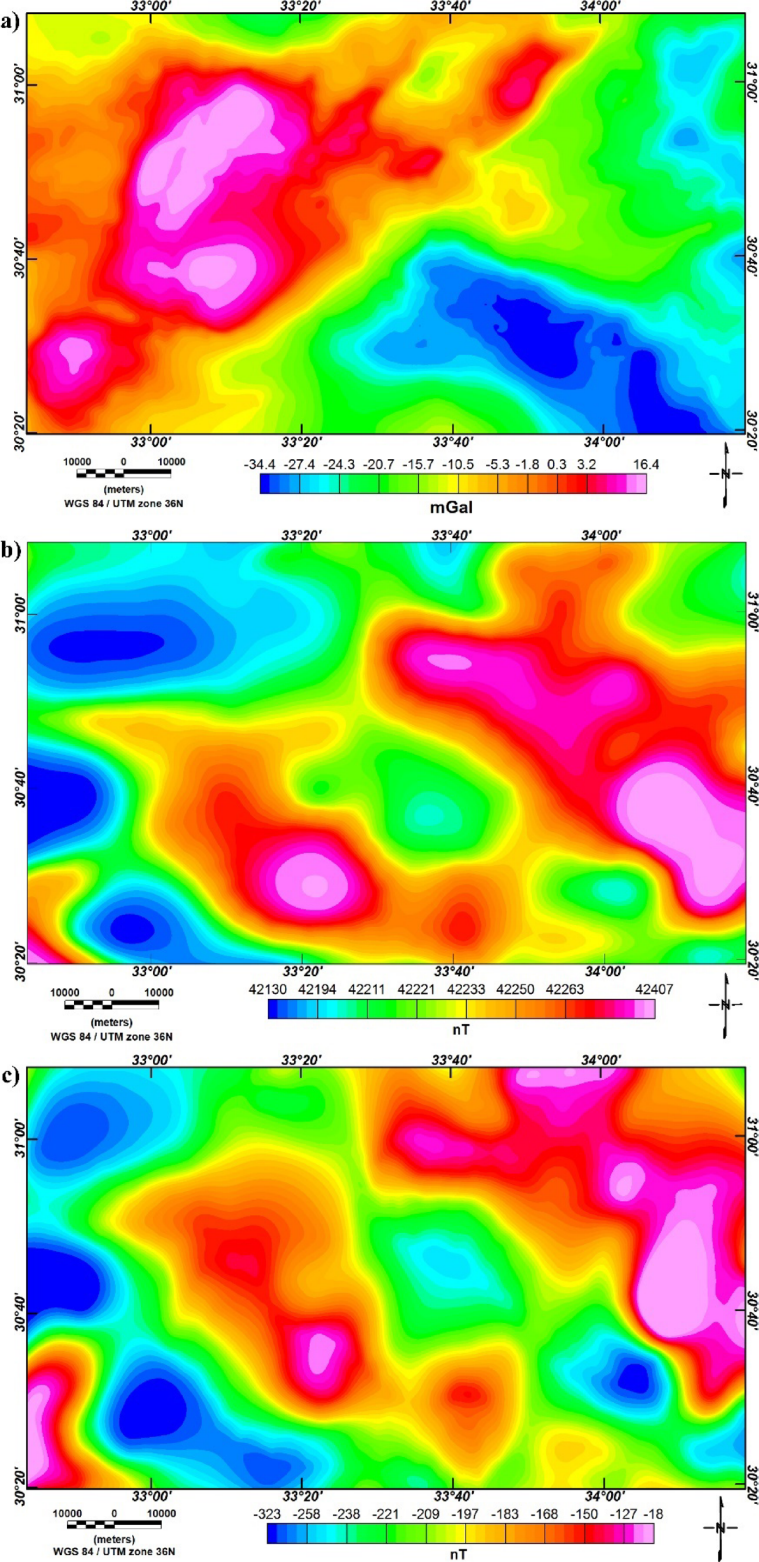
(**a**) Bouguer gravity (BG) map, (**b**) TMA map, and (**c**) RTP map of Northern Sinai, Egypt.

The BG map of northern Sinai, Egypt, is presented in Fig. 9a, which shows density highs in the western part and lows in the eastern part of the area. The obtained total magnetic anomaly (TMA) of the study region is shown in Fig. 9b that is reduced-to-pole (RTP) according to^82^ (Fig. 9c).

**Fig. 10:**
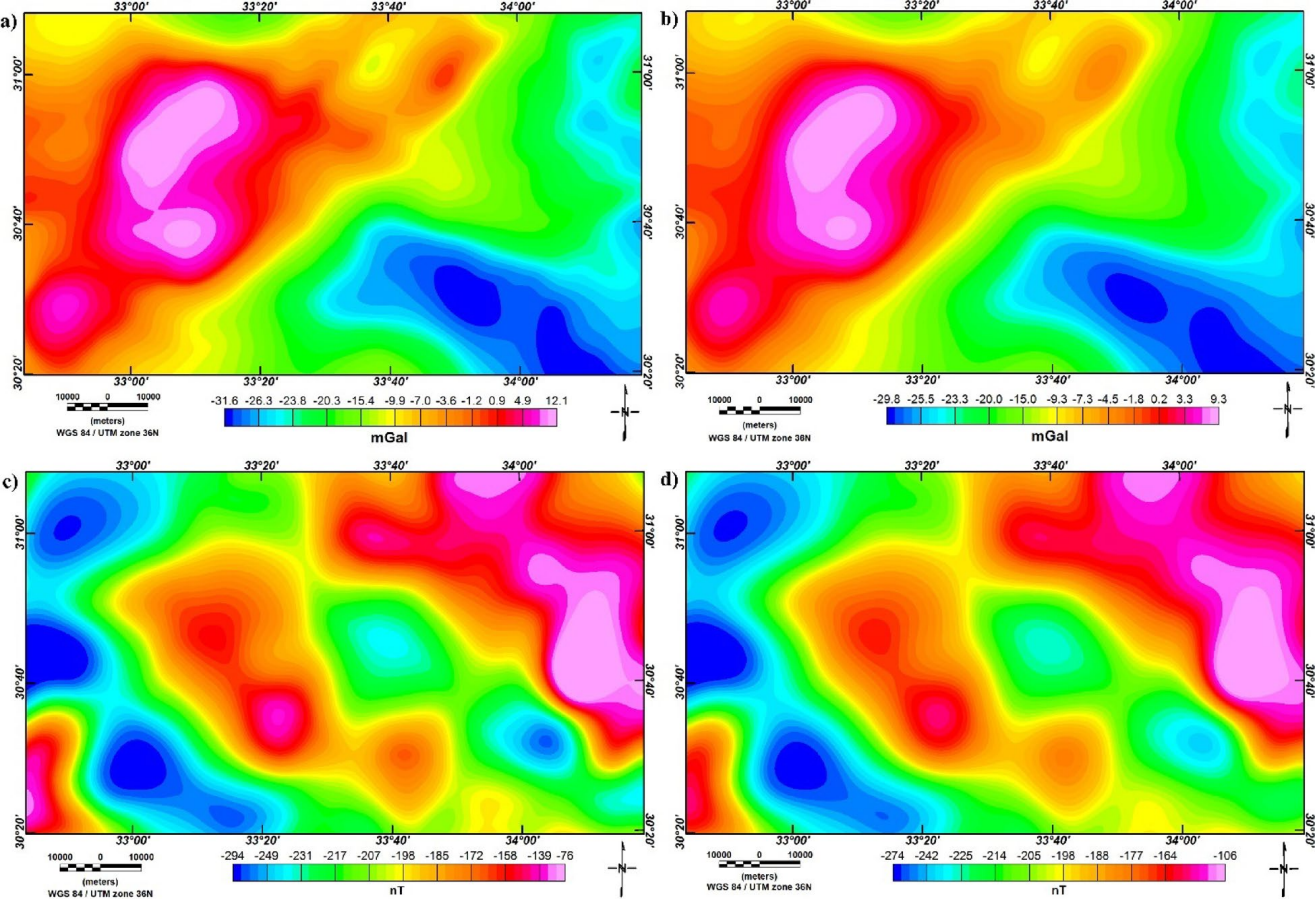
(**a**) UC-BG map at altitude of 2 km, (**b**) UC-BG map at altitude of 4 km, (**c**) UC-RTP map at altitude of 2 km, and (**d**) UC-RTP map at altitude of 4 km.

Moreover, the BG and RTP data are upward continued (UC) to levels of 2, and 4 km (Fig. 10) to follow and delineate the changes in density and magnetization of structures downward^8,83–85^.

**Fig. 11:**
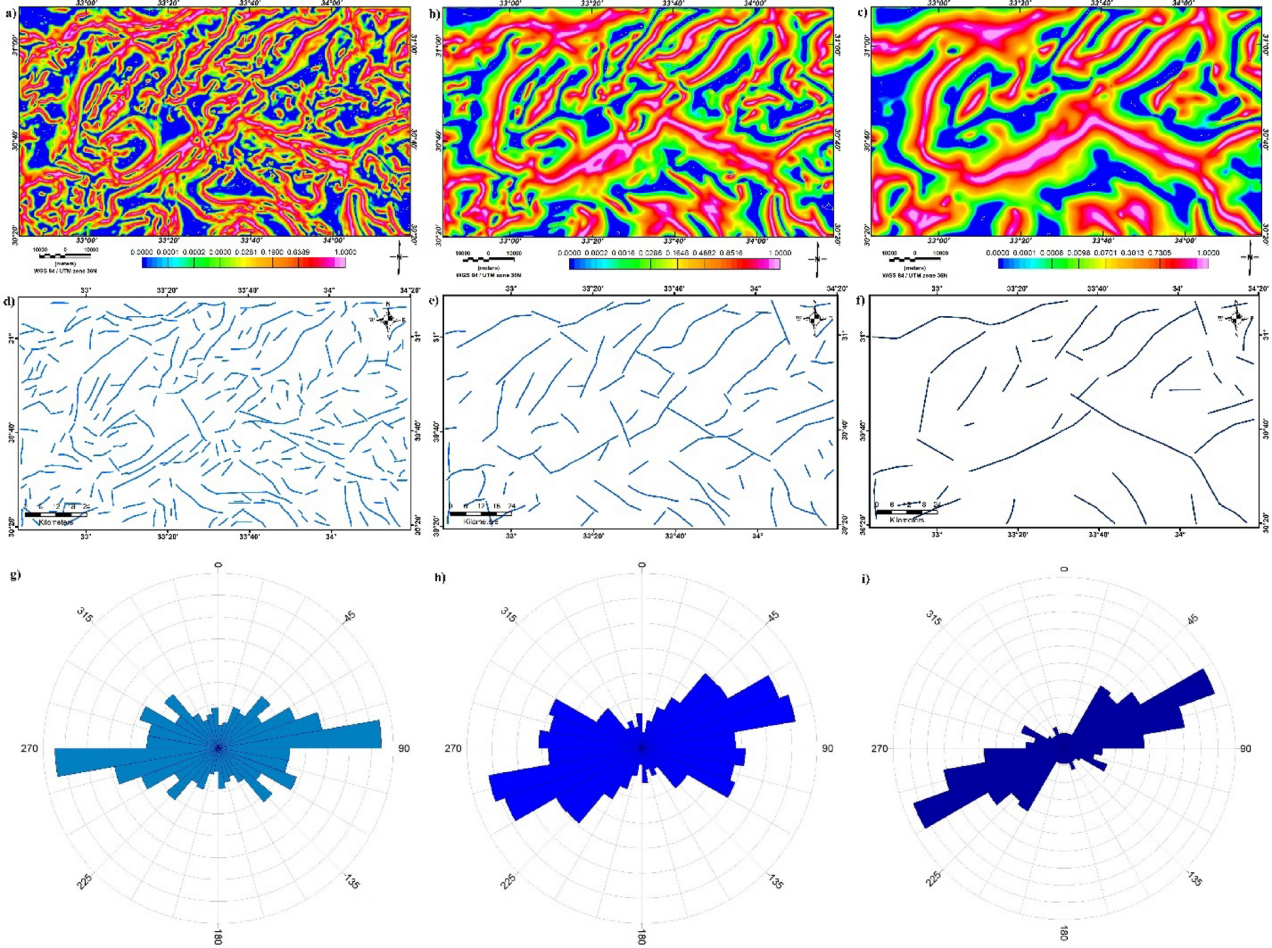
(**a**) LSF map of BG, (**b**) LSF map of UC-BG at 2 km altitude, (**c**) LSF map of UC-BG at 4 km altitude, (**d**) Lineaments of LSF-BG, (**e**) Lineaments of LSF-UC-BG at 2 km, (**f**) Lineaments of LSF-UC-BG at 4 km, (**g**) Rose diagram of LSF-BG, (**h**) Rose diagram of LSF-UC-BG at 2 km, and (**i**) Rose diagram of LSF-UC-BG at 4 km.

The LSF technique is applied to BG and UC of BG at altitudes of 2 and 4 km (Fig. 11a and b, and c, respectively). The Traced lineaments are shown in Figs. 8e and 11d, and f and statistically analyzed using rose diagrams (Fig. 11g and h, and i). It can be noticed that the E-W, ENE, and WNW are the main dominant trends at shallow depths in addition to the presence of other directions as NW and NE (Fig. 11a and d, and g). At 2 km, the ENE, NE, WNW, and E-W are dominant (Fig. 11b and e, and h) while the prevailing structures at 4 km are the ENE, NW, and E-W with minor traces of WNW and NW directions (Fig. 11c and f, and i).

**Fig. 12:**
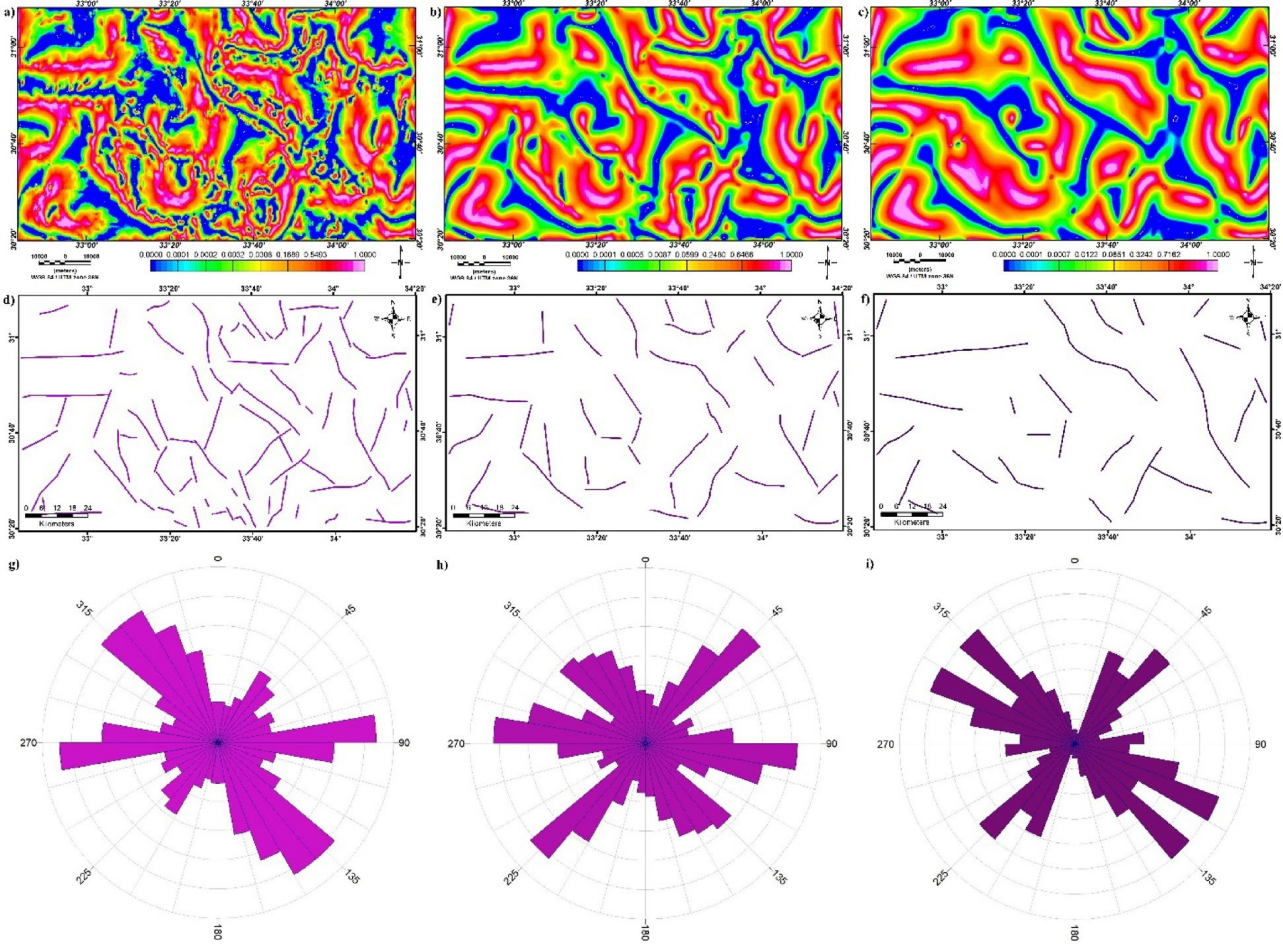
(**a**) LSF map of RTP, (**b**) LSF map of UC-RTP at 2 km altitude, (**c**) LSF map of UC-RTP at 4 km altitude, (**d**) Lineaments of LSF-RTP, (**e**) Lineaments of LSF-UC-RTP at 2 km, (**f**) Lineaments of LSF-UC-RTP at 4 km, (**g**) Rose diagram of LSF-RTP, (**h**) Rose diagram of LSF-UC-RTP at 2 km, and (**i**) Rose diagram of LSF-UC-RTP at 4.

On the other hand, the LSF is used to delineate lineaments and structures of RTP and UC of RTP at altitudes of 2 and 4 km (Fig. 12a and b, and c, respectively). The outlined structural features are illustrated in Fig. 12d and e, and f and statistically investigated employing rose diagrams (Fig. 12g and h, and i). The main structural trends obtained from LSF-RTP are the NW, E-W, NE, and ENE. The LSF of UC-RTP at 2 km revealed that the ENE, NE, and NW are dominant (Fig. 12b and e, and h). While Fig. 12c and f, and i represent that the main structural features controlling the Northern Sinai area at a depth of 4 km are the WNW, NW, NE, NNE, and E-W directions.

## Discussion

This study integrates remote sensing, gravity, and magnetic datasets to unravel the structural architecture of Northern Sinai, which represents one of the most tectonically active regions along the northeastern margin of Africa. The application of the newly introduced Logsigm Function (LSF) filter to synthetic models demonstrates its exceptional capability in detecting overlapping or closely spaced geological boundaries. Unlike traditional edge detection methods, the LSF filter maintains resolution at depth and accurately delineates both shallow and deep features, even in the presence of noise. When applied to real data from Northern Sinai, the LSF proved highly effective in mapping structural elements in both the sedimentary cover and basement rocks.

Surface structural trends extracted from enhanced DEM and multispectral satellite imagery reveal dominant NE–SW and N–S orientations. These align well with regional tectonic frameworks associated with the Syrian Arc Fold Belt. However, subsurface structures derived from Bouguer gravity and magnetic data reveal more complex trends, particularly at depth, where ENE, E–W, and NW directions become more prominent. This vertical variation highlights structural segmentation with depth and underscores the role of reactivated basement faults that are not always evident at the surface.

The differing trends observed in the LSF-filtered gravity and magnetic datasets (Figs. 11 and 12) reflect the inherent sensitivity of each geophysical method to different physical properties and depths. Gravity data, responsive to density contrasts, excels in identifying deep-seated structures and crustal discontinuities often linked to major tectonic processes. Conversely, magnetic data, sensitive to variations in magnetization, better resolves shallower crustal features such as dykes, faults, and magnetized intrusions. These differences are expected in structurally complex regions like Northern Sinai, where multiple tectonic phases have left a multilayered geophysical imprint^1,86–90^. Rather than contradictory, the trends extracted from gravity and magnetic data using the LSF filter are complementary, together offering a more complete vision of the subsurface framework. Importantly, the surface lineaments automatically extracted from the enhanced DEM and Landsat imagery were cross-validated against manually interpreted structures derived from high-resolution optical imagery. This comparison, especially across the key exposures of Gebel Halal, Yelleg, and Maghara, confirmed the reliability of the automated lineament extraction and enhanced confidence in the interpretation of subsurface structural trends.

The predominance of ENE, E–W, and WNW trends in gravity-derived data further emphasizes the effectiveness of gravity in imaging deep-seated structures, especially beneath thick sedimentary cover. Unlike magnetic methods, which may be hindered by weak magnetization in overlying sediments, gravity techniques remain sensitive to variations in density, making them especially useful for detecting concealed faults and intra-sedimentary structures^1,2^.

The vertical variation in structural trends also reflects the mechanical decoupling between sedimentary cover and the underlying basement. This decoupling is a hallmark of tectonic inversion settings such as the Syrian Arc system, where extensional Mesozoic faults were later reactivated during Late Cretaceous to Paleogene compression. The structural configuration of Gebel Halal, Gebel Maghara, and Gebel Yelleg aligns with this inversion model, as confirmed by the patterns identified in the current study.

The observed vertical variation in structural trends, along with the evidence for reactivated basement faults from gravity and magnetic data, points toward a thick-skinned deformation style in Northern Sinai. The alignment of deeper ENE to E-W faults with inherited basement fabrics, as imaged in gravity data, and their decoupling from NE–SW and N–S surface trends extracted from remote sensing, reflects a strong mechanical contrast between the cover and basement. This supports a deformation regime where tectonic forces were transmitted into the crystalline basement, reactivating pre-existing structures during multiple tectonic phases. Such a thick-skinned style is consistent with regional tectonic models of the Syrian Arc System, where Mesozoic extensional faults were inverted during Late Cretaceous-Paleogene compression, affecting both the cover and the underlying basement. This interpretation is reinforced by the tectonic configuration of key structures like Gebel Halal, Maghara, and Yelleg, which show signs of inversion tectonics with basement-involved faulting.

The success of the LSF filter lies in its combination of mathematical simplicity, robustness, and edge-sharpening capacity. Its application extends beyond academic interpretation:

- It can enhance mineral exploration by delineating ore-controlling structures.
- It supports hydrocarbon exploration by identifying faults and folds acting as structural traps.
- It contributes to crustal-scale studies by outlining major terrane boundaries.

## Conclusions

This study presents robust edge detection approach (LSF) that significantly enhances the interpretation of potential field data for delineating geological structures. Through synthetic modeling, the LSF demonstrated superior capability in accurately identifying closely spaced and overlapping source boundaries, while maintaining stability in the presence of noise. Its mathematical simplicity and high spatial resolution make it an effective and computationally efficient tool for geophysical edge detection.

Application of the LSF filter to real gravity and magnetic data from Northern Sinai revealed major structural features across different depth levels, including ENE, E-W, and NW-trending faults and folds that align with known tectonic frameworks. Importantly, the filter also identified previously unrecognized lineaments, contributing new insights into the tectonic evolution of this inverted basin system.

To validate and contextualize subsurface results, remote sensing techniques (including enhanced Landsat 8 OLI and PALSAR DEM data) were used to extract and analyze surface lineaments. The correlation between automatically extracted and manually interpreted structures, especially across key exposures such as Gebel Halal, Yelleg, and Maghara, reinforces the reliability of the remote sensing results. When integrated with LSF-filtered geophysical data, these surface observations reveal a clear vertical differentiation in structural patterns, suggesting deformation decoupling and the influence of inherited basement structures.

Overall, this multidisciplinary approach confirms the utility of the LSF method in structurally complex settings, offering reliable results for both shallow and deep interpretations. The combined use of remote sensing and potential field data allows for a more comprehensive structural framework, essential for understanding tectonic processes and supporting mineral exploration.

## Data Availability

Data sets generated during the current study are available from the corresponding author on reasonable request.
